# Single-incision total laparoscopic hysterectomy

**DOI:** 10.4103/0972-9941.72389

**Published:** 2011

**Authors:** Rakesh Sinha, Meenakshi Sundaram, Chaitali Mahajan, Shweta Raje, Pratima Kadam, Gayatri Rao, Prachi Shitut

**Affiliations:** Department of Gynaecological Endoscopy, BEAMS Hospital, 674, 16^th^ Cross Road, Behind Khar Gymkhana, Khar Pali, Mumbai 400052, India

**Keywords:** E-NOTES, single-incision laparoscopic surgery, single-port surgery, SILS, transumbilical surgery

## Abstract

Single-incision laparoscopic surgery is an alternative to conventional multiport laparoscopy. Single-access laparoscopy using a transumbilical port affords maximum cosmetic benefits because the surgical incision is hidden in the umbilicus. The advantages of single-access laparoscopic surgery may include less bleeding, infection, and hernia formation and better cosmetic outcome and less pain. The disadvantages and limitations include longer surgery time, difficulty in learning the technique, and the need for specialized instruments. Ongoing refinement of the surgical technique and instrumentation is likely to expand its role in gynecologic surgery in the future. We perform single-incision total laparoscopic hysterectomy using three ports in the single transumbilical incision.

## INTRODUCTION

Minimally invasive surgery has substantially decreased the length of hospital stay, decreased the need for postoperative analgesia, and improved the recovery time. However, few modifications have been made in peritoneal access in the last decade that would lead to further improvement in postoperative recovery and cosmesis. Single-incision laparoscopic surgery is also referred to as embryonic natural orifice transumbilical endoscopic surgery (E-NOTES).[1] Conventional laparoscopy uses three or four ports to complete a gynecologic procedure. The primary advantage of the current approach is limiting the port incisions to one site, the umbilicus. Therefore, the surgical scar can be hidden within the umbilicus, rendering the surgery virtually “scarless.” Laparoscopic tubal ligation using single-port access was described as early as 1973.[2]

Single-port access (SPA) laparoscopic surgery has been reported for appendectomies, cholecystectomies, and several urology procedures.[3] In addition, single-port hysterectomy was also reported in the early 1990s.[4] We describe our method of performing single-incision total laparoscopic hysterectomy with endosuturing.

## OPERATIVE PROCEDURE

Preoperatively, all the patients are kept on liquid diet for a day and bowel preparation is done with Exelyte solution. All the surgeries are performed under general anesthesia with the patient in modified lithotomy position. The Veress needle is inserted at the umbilicus in most patients and in selected patients with previous surgeries, the Palmer’s point is used. The Palmer’s point[5] (a point 3 cm below the left costal margin in the midclavicular line) is a safe zone in all patients other than those with splenomegaly.

**Figure 1:**
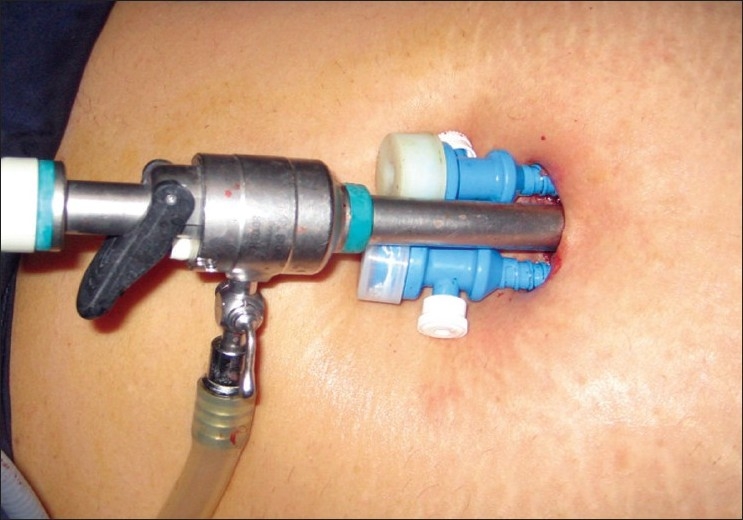
Single intraumbilical incision

**Figure 2:**
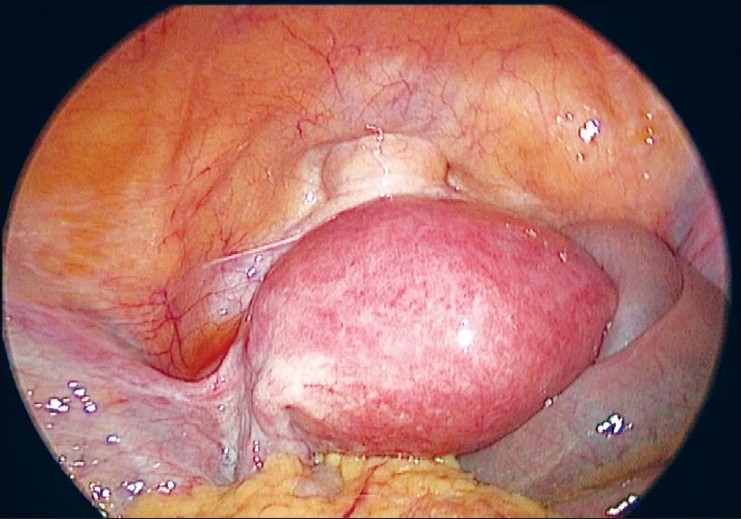
Single-incision one 10 mm and two 5 mm ports

After insufflations with carbon dioxide, a 2 cm incision is made at the lower margin of the umbilicus. A 10 mm trocar is introduced into the incision. We always prefer to use the 30 degree telescope for advanced laparoscopic procedures. Two 5 mm accessory trocars are introduced on either side of the optical trocar [Figures 1 and 2].

**Figure 3:**
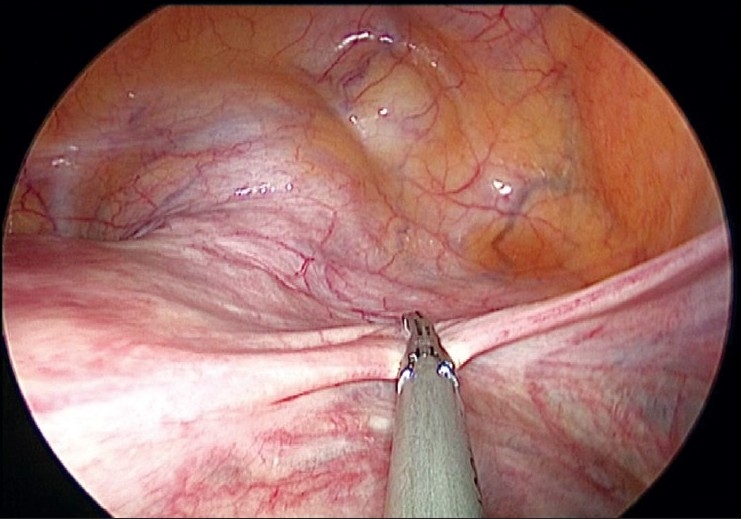
Uterus bulky

**Figure 4:**
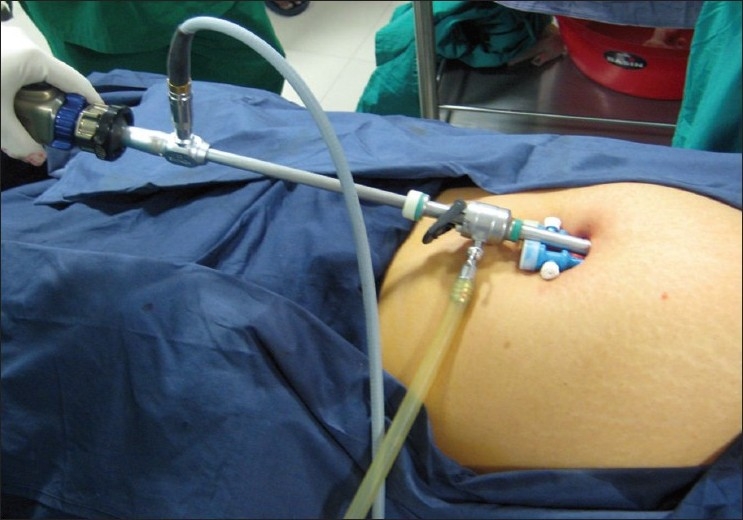
Right round ligament desiccated and cut with harmonic

**Figure 5:**
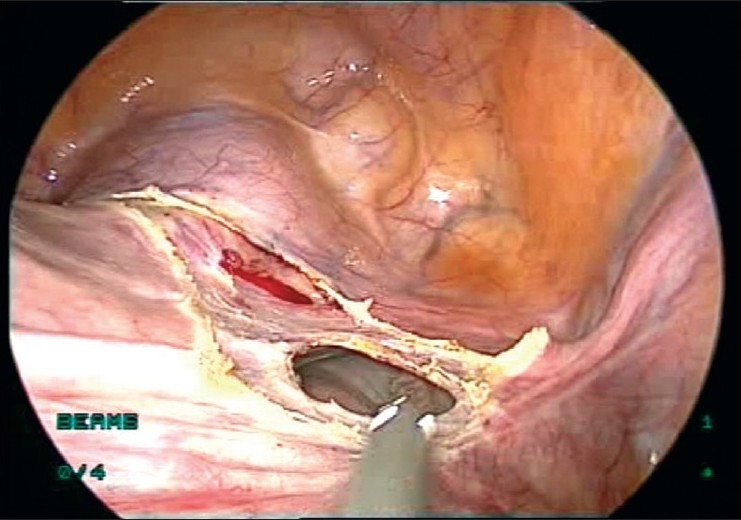
Window made in broad ligament

**Figure 6:**
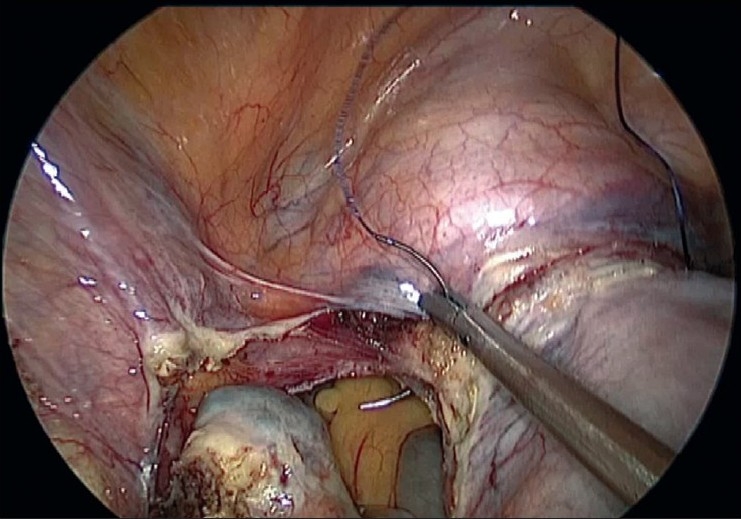
Left uterine artery sutured

**Figure 7:**
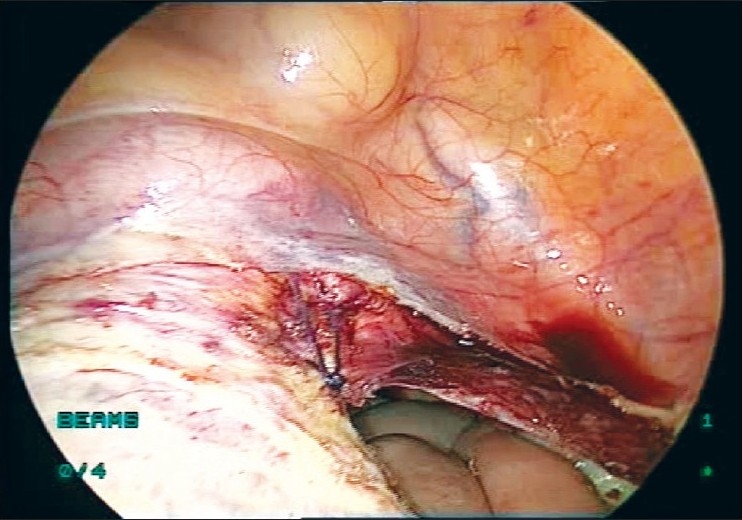
Right uterine artery sutured

The uterus is manipulated by a 5 mm myoma spiral from the accessory port [Figure 3]. We start the dissection from the right round ligament. The harmonic ultracision is used to desiccate and cut the right round ligament [Figure 4]. The uterovesical fold of the peritoneum is identified and opened from the round ligament on either side. The bladder is dissected down completely so that the uterine vessels on either side can be clearly seen. A window is created in the broad ligament close to the uterine vessels [Figure 5]. This helps visualization of the posterior aspect and prevents accidental suture placement through bowel loops. The ascending branch of the uterine artery is identified close to the isthmus. The uterine vessels are ligated at this level close to the uterus by transfixation using 1-0 delayed absorbable suture material [Figures 6 and 7].

**Figure 8:**
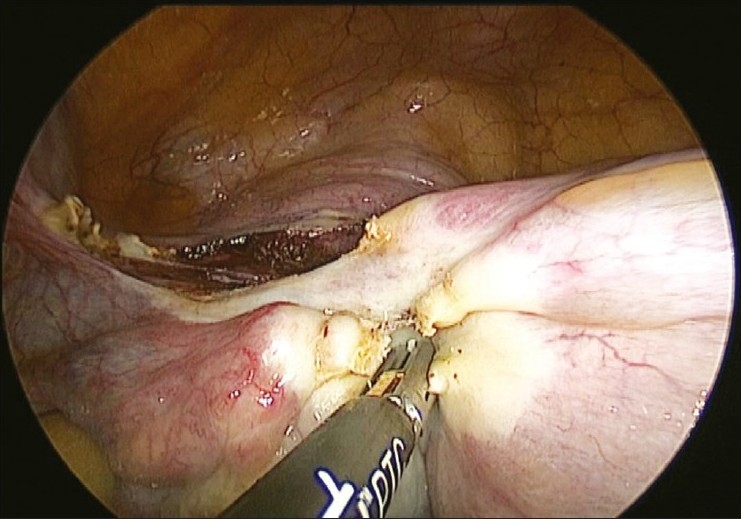
Left cornuals desiccated and cut

**Figure 9:**
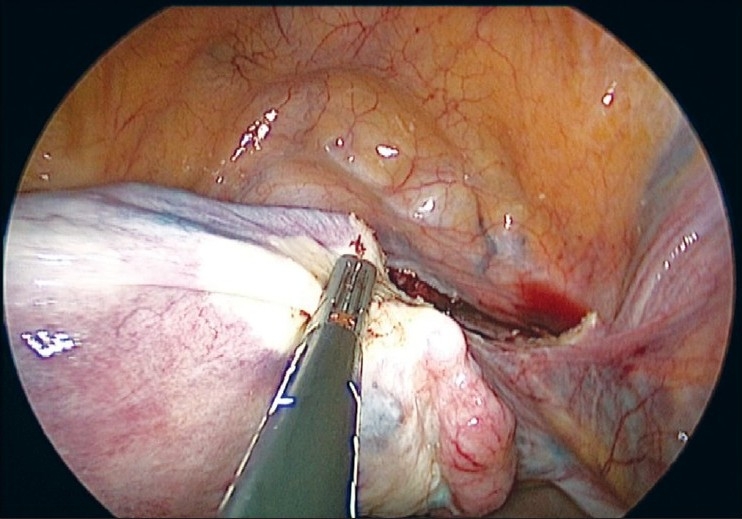
Right cornuals desiccated and cut

**Figure 10:**
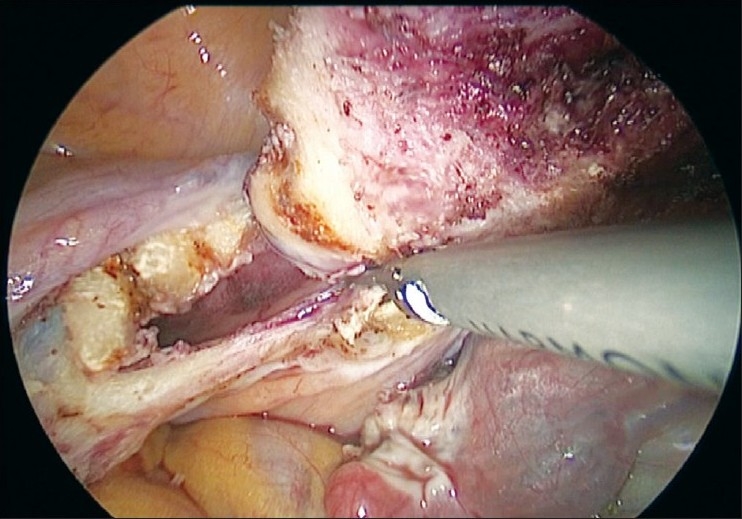
Vaginal vault opened

It is rather challenging for the surgeon to suture the uterine arteries laparoscopically through the single-incision ports. Skill and expertise is essential to accomplish this suturing. The cornual pedicles are then desiccated and cut either using vessel sealing device or the harmonic ultracision [Figures 8 and 9]. The ligated uterine pedicles are cut. The uterosacrals and cardinal ligaments are desiccated and cut with the vessel sealing device or the harmonic ultracision. The vaginal vault is opened from one side [Figure 10]. The position of the myoma spiral is then changed so that the opposite side pedicles can be taken. The cornual, uterine, and uterosacral aspects of the other side are taken in a similar way and the vaginal vault is opened. The specimen is detached completely.

**Figure 11:**
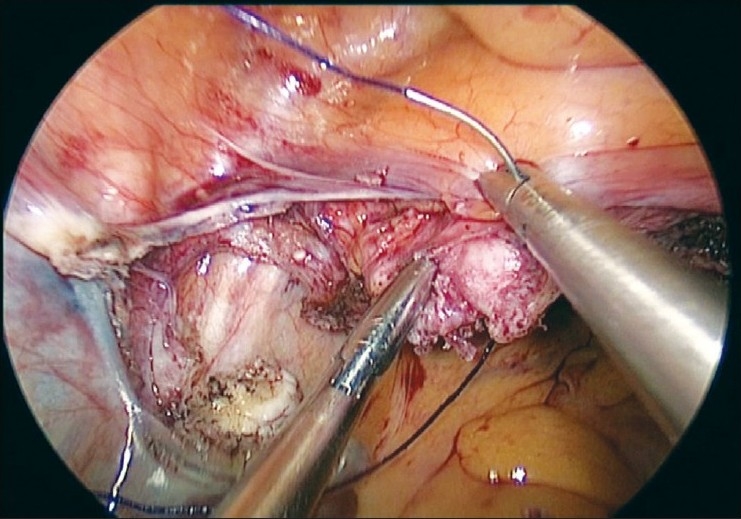
Vault sutured laparoscopically

**Figure 12:**
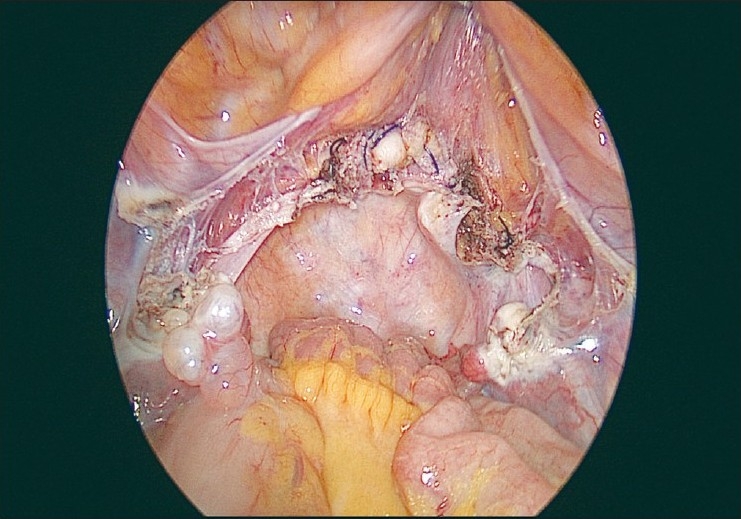
Final view

The uterus is removed vaginally when feasible. In cases where the size of the uterus is large for vaginal delivery, a morcellator is introduced through the vault opening and the specimen is retrieved by morcellation with the claw forceps. This is done totally under laparoscopic guidance. If both ovaries need to be removed, the infundibulopelvic ligaments are desiccated and cut, and the ovaries are delivered vaginally. The vaginal vault is then sutured laparoscopically with No. 1 delayed absorbable interrupted figure-of-eight sutures [Figures 11 and 12].

**Figure 13:**
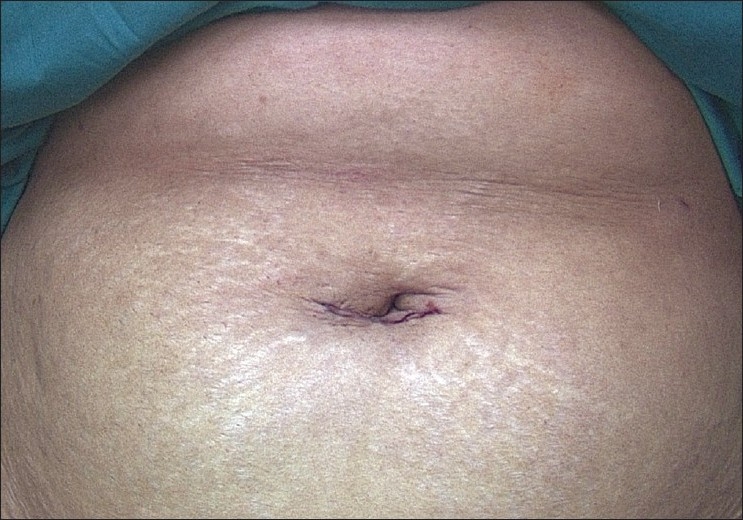
Skin closed with subcuticular sutures

In cases of previous caesarean sections where the bladder is densely adherent, we start from the left cornual structures. The uterovesical fold is opened and the bladder is dissected down by the lateral approach. A definitive plane above the level of the uterine vessels can be identified between the cervix and the bladder by this approach and the bladder is dissected down. The uterine vessels are then ligated on either side and the dissection is completed. The single-incision is closed with subcuticular sutures using No 3-0 delayed absorbable suture [Figure 13]. This standard technique of total laparoscopic hysterectomy is performed in all cases with single-incision. However, in the case of very large myomas that render the procedure very difficult, certain modifications to the above technique may have to be adopted.

## DISCUSSION

Since the first laparoscopic hysterectomy was reported by Reich *et al* in 1989,[6] the trend has been to change the hysterectomy approach from the open abdominal to a laparoscopic technique. The latest innovations in the field of minimally invasive surgery are to further reduce the morbidity associated with laparoscopic surgery and to improve cosmetic outcomes. Single-incision surgery has been reported to offer patients improved cosmetic outcomes as compared with multiport laparoscopic surgery, and possibly less postoperative pain,[7] although these potential benefits have yet to be demonstrated in a well-designed prospective trial. We in our center have been doing single-incision surgeries for total laparoscopic hysterectomy, laparoscopic myomectomy, and laparoscopic ovarian cyst excision.

However, there are several limitations to this approach, including lack of triangulation, instrument crowding at the umbilicus, and difficulty with suturing using traditional sutures. It may be challenging to apply single-port surgery to more complex disorders such as very large myomas and severe pelvic adhesive disease.

Lee *et al*[8] have described single-port access laparoscopic-assisted vaginal hysterectomy using wound retractor and glove in 24 patients and concluded that it was safe and effective, and the procedure could be learned over a short period of time. Yoon *et al*[9] evaluated the feasibility, safety, and operative outcome of management of myomas and adenomyosis using single-port access subtotal hysterectomy with transcervical morcellation using a wound retractor and a surgical glove. Their study concluded that single-port access subtotal hysterectomy is safe and effective and results in almost no visible scar. With more experience and advanced instruments, this surgical procedure can offer a safe and effective option to hysterectomy with an excellent cosmetic outcome.

Langebrekke and Qvigstad[10] described total laparoscopic hysterectomy through a single-port without vaginal surgery. They have reported vaginal closure using bidirectional barbed sutures. The technique of total laparoscopic hysterectomy is possible with single access; however, most surgeons find the suture technique difficult through a single port. Compared with traditional sutures, the benefits of the bidirectional self-retaining sutures with tissue retainers (barbs) include speed and economy of suture placement. There is no need of suture knotting, and by not using multiple suture loops, the tension can be more uniformly distributed along the length of the vaginal cuff. We have also used barbed sutures for closing the vaginal cuff in some patients. Using improved instruments and bidirectional self-retaining sutures, the laparoscopic technique is almost as easy to perform as with the traditional four-port access.

In conclusion, single-incision total laparoscopic hysterectomy is feasible in selected cases and is a safe cosmetic alternative to conventional multiport laparoscopy. Currently, careful case selection is important so that these procedures can be explored safely, with a low threshold to convert to standard laparoscopy as indicated for safety and quality of care.
